# Causality within the Epileptic Network: An EEG-fMRI Study Validated by Intracranial EEG

**DOI:** 10.3389/fneur.2013.00185

**Published:** 2013-11-14

**Authors:** Anna Elisabetta Vaudano, Pietro Avanzini, Laura Tassi, Andrea Ruggieri, Gaetano Cantalupo, Francesca Benuzzi, Paolo Nichelli, Louis Lemieux, Stefano Meletti

**Affiliations:** ^1^Department of Biomedical Sciences, Metabolism, and Neuroscience, NOCSE Hospital, University of Modena and Reggio Emilia, Modena, Italy; ^2^Department of Clinical and Experimental Epilepsy, UCL Institute of Neurology, National Hospital for Neurology and Neurosurgery, London, UK; ^3^Department of Neuroscience, University of Parma, Parma, Italy; ^4^“C. Munari” Epilepsy Surgery Center, Niguarda Hospital, Milan, Italy; ^5^Department of Life and Reproduction Sciences, University of Verona, Verona, Italy

**Keywords:** functional neuroimaging, epilepsy surgery, seizure onset zone, intracerebral recordings, effective connectivity, EEG, fMRI, dynamic causal modeling

## Abstract

Accurate localization of the Seizure Onset Zone (SOZ) is crucial in patients with drug-resistance focal epilepsy. EEG with fMRI recording (EEG-fMRI) has been proposed as a complementary non-invasive tool, which can give useful additional information in the pre-surgical work-up. However, fMRI maps related to interictal epileptiform activities (IED) often show multiple regions of signal change, or “networks,” rather than highly focal ones. Effective connectivity approaches like Dynamic Causal Modeling (DCM) applied to fMRI data potentially offers a framework to address which brain regions drives the generation of seizures and IED within an epileptic network. Here, we present a first attempt to validate DCM on EEG-fMRI data in one patient affected by frontal lobe epilepsy. Pre-surgical EEG-fMRI demonstrated two distinct clusters of blood oxygenation level dependent (BOLD) signal increases linked to IED, one located in the left frontal pole and the other in the ipsilateral dorso-lateral frontal cortex. DCM of the IED-related BOLD signal favored a model corresponding to the left dorso-lateral frontal cortex as driver of changes in the fronto-polar region. The validity of DCM was supported by: (a) the results of two different non-invasive analysis obtained on the same dataset: EEG source imaging (ESI), and “psycho-physiological interaction” analysis; (b) the failure of a first surgical intervention limited to the fronto-polar region; (c) the results of the intracranial EEG monitoring performed after the first surgical intervention confirming a SOZ located over the dorso-lateral frontal cortex. These results add evidence that EEG-fMRI together with advanced methods of BOLD signal analysis is a promising tool that can give relevant information within the epilepsy surgery diagnostic work-up.

## Introduction

The objective of this clinical study was to investigate the causal relationships, by means of dynamic causal modeling (DCM) on fMRI data, between brain areas showing IED-related blood oxygenation level dependent (BOLD) changes in a patient with drug-resistant epilepsy prior to surgery in comparison to the results of intracranial EEG recording (icEEG) and in light of post-surgical outcome. A multi-modal approach of fMRI and EEG data analysis has been applied to verify the DCM results. We aim to show the applicability of the DCM method on fMRI data for the identification of the seizure onset zone (SOZ) and the epileptic propagation networks. We will then discuss the potential usefulness of such methodology within the epilepsy surgery diagnostic work-up. Our work, although limited to a single subject, might hence represent a “proof of concept” study aimed to provide evidences in favor of this non-invasive tool in the management of patients with focal epilepsies candidate to surgery.

### Case presentation

We studied a 27-year-old left-handed man. Seizures started at the age of 5 months in the form infantile spasms, which were controlled with benzodiazepines and steroids. One year later, brief right hemi-clonic seizures recurred, that remitted with a pulse corticosteroid treatment. After a prolonged seizure-free period, seizures relapsed with a frequency of 2–3 times/week. Seizures were characterized by motor arrest, staring, flushing; then laughing, bimanual automatisms, repetitive left foot movements with oro-alimentary automatisms followed. Secondary generalized tonic-clonic seizures occurred about once a month. His past medical history, including birth and development milestones, was unremarkable. Neurological examination was normal.

**Figure 1:**
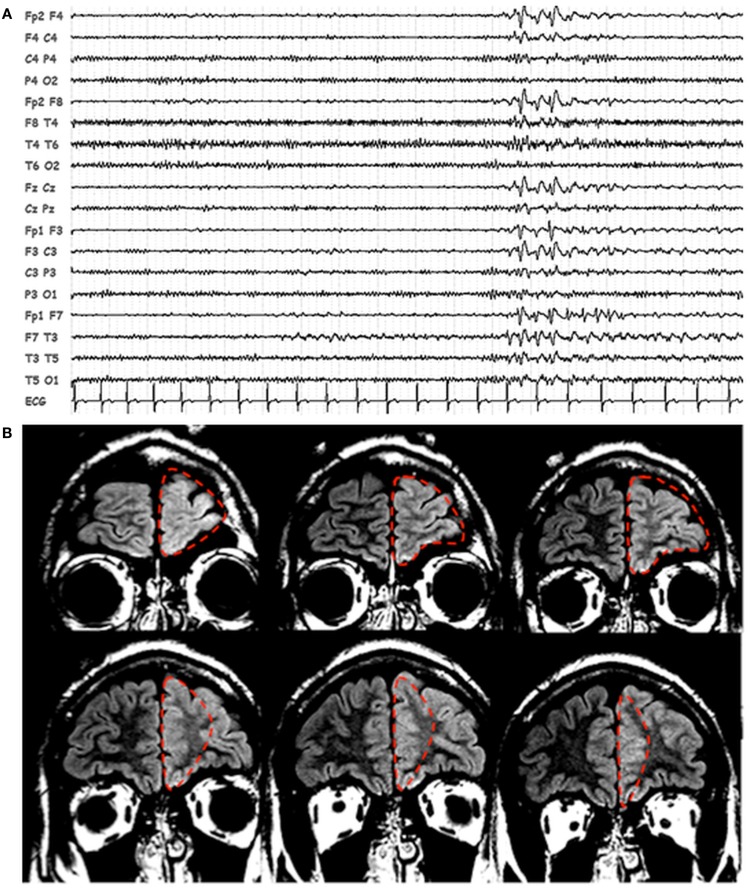
**Patients’ EEG and structural MRI scan**. **(A)** Representative page of scalp EEG interictal epileptiform abnormalities with bi-frontal/left frontal predominance. EEG is displayed in bipolar montage. **(B)** Fluid attenuated inversion recovery (FLAIR), coronal images showing thickening and blurring of the left prefrontal cortex. The dashed red line identified the boundaries of the first surgery resection.

Scalp EEG revealed interictal bilateral (left predominant) frontal spikes and spikes-and-waves (Figure 1A), while prolonged video-EEG recordings showed a left fronto-temporal seizure onset. Structural MRI (Philips, 3T) revealed thickening and blurring of the left fronto-polar cortex (LFp) suggesting the presence of a focal cortical dysplasia (Figure 1B). Based on non-invasive electro-clinical findings, particularly considering the blurring of the fronto-polar cortex, a tailored cortectomy limited to this frontal lobe region was performed (Figure 1B). Pathology confirmed the presence of focal cortical dysplasia (Type IIb). Three months after surgery, seizures relapsed characterized by the same stereotyped behavioral sequences: motor arrest and staring were the first ictal symptoms, followed by bimanual/pedal automatic behavior. The only difference was the absence of laugh (which has never been seen as initial ictal symptom in the pre-surgical seizures). During the post-surgical follow-up period (3 years), seizures recurred in clusters one at month and were generally of shorter duration respect with the pre-surgery seizures.

### EEG-fMRI data acquisition and conventional analysis

Within the pre-surgical assessment, an EEG/fMRI study was performed in order to identify the IED-related hemodynamic changes. The recording was performed in the early afternoon and sedation was not used. The Human Ethic Committee of the University of Modena and Reggio Emilia, Italy granted approval. Written informed consent was obtained from the patient.

Scalp EEG has been recorded by means of a 32-channel MRI-compatible EEG recording system (Micromed, Mogliano Veneto, Italy). Electrodes were placed according to conventional 10–20 locations. Prior to in-magnet EEG recording, 30 min out-of magnet EEG was collected in a room adjacent to the scanner. Foam pads were used to help secure the EEG leads, minimize motion, and improve the patient’s comfort. Data were transmitted via an optic fiber cable from the amplifier (1.024 Hz sampling rate) to a computer located outside the scanner room. To avoid saturation, the EEG amplifiers have a resolution of 22 bits with a range of ±25.6 mV.

The patient was constantly observed and recorded by means of a small camcorder positioned on the head coil inside the scanner pointing to the patient’s face to obtain a split-screen video-EEG documentation during the fMRI recording. The patient was asked to rest with eyes closed and keep still during fMRI acquisitions.

Functional data have been acquired using Philips Intera system at 3T and a gradient-echo planar sequence from 30 axial contiguous slices (TR = 3.000 ms; in-plane matrix = 64 × 64; voxel size: 4 × 4 × 4) over three 10-min sessions (200 volumes/session) with continuous simultaneous EEG recording. A high-resolution T1-weighted anatomic image has been acquired to allow accurate anatomic localization of activations/deactivations. The volume consisted of 170 sagittal slices (TR = 9.9 ms; TE = 4.6 ms; in-plane matrix = 256 × 256; voxel size = 1 mm × 1 mm × 1 mm).

Off-line analysis of the EEG was performed by means of the BrainQuick System Plus software (Micromed, Mogliano Veneto, Italy), including the correction of the gradient artifacts (1) and filtering of the EEG signal. In addition, the EEG data were exported in the .edf format and reviewed and analyzed by means of the BrainVision Analyzer 2.0 software (Brain Products, Munich, Germany). A bandpass filter between 1 and 70 Hz was applied to the continuous recording and channels showing high impedance or electrode displacement artifacts were interpolated through a cubic spline. EEG Independent Component Analysis (ICA) (2, 3) was applied in order to separate the generators of EEG activities and maximizing the statistical independence among them. To optimize artifactual activities removal, blinks, and saccades were marked on channel Fp1; R-peaks due to cardiac artifact were also marked for subsequent artifact removal when present. Two experienced electroencephalographers (Stefano Meletti, Anna Elisabetta Vaudano) reviewed the pre-processed EEG recordings independently in order to identify the IED and to compare their features with the ones observed during the long-term out-scanning video-EEG monitoring.

The Matlab 7.1 and SPM8 (Welcome Department of Imaging Neuroscience, London, UK) software was used for fMRI data analysis. All functional volumes were slice time corrected, realigned to the first volume acquired, and smoothed with 8 mm × 8 mm × 8 mm full-width half maximum (FWHM) Gaussian Kernel. The six motion parameters derived from the fMRI pre-processing (translation and rotation in the *X, Y*, and *Z* direction, respectively) were used as covariates in the general linear model (GLM). IED were visually marked and served as onsets for a GLM convolved with the standard hemodynamic response function (HRF). IED were considered as stick functions or blocks with variable duration as appropriate. One-tailed *t*-test was applied to test for regional BOLD increases or decreases in relationship to the IED. The computed SPM{T} was thresholded at *p* < 0.05, corrected for multiple comparisons. The statistical parametric *t*-maps were superimposed on the co-registered patient’s anatomical MRI scans for localization purposes.

**Figure 2:**
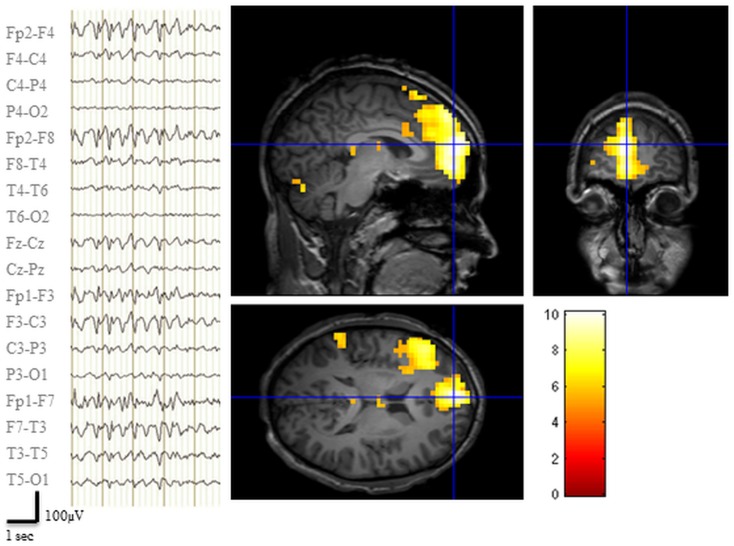
**Pre-surgical EEG-fMRI study**. Left panel: representative scalp EEG page recorded during scanning after off-line artifact subtraction. BrainQuick System Plus software (Micromed) was used for off-line correction of the gradient artifacts and filtering of the EEG signal. EEG trace (displayed in bipolar montage) shown bi-frontal IED (total: 142) with left predominance. Right panel: results of canonical GLM, SPM{T} (*p* < 0.05 corrected for multiple comparison) showing BOLD signal increases IED correlated, crosshair at the global statistical maximum: activations were observed at the left dorso-lateral frontal cortex, the ipsilateral frontal pole plus a small cluster in the ipsilateral temporo-parietal cortex. Deactivations were evident bilaterally in precuneus and dorsal parietal areas (data not shown). Results were overlaid on patient’s T1 scan.

The EEG recorded during the pre-surgical fMRI session revealed 142 bi-frontal IED (Figure 2). Two prominent clusters of significant BOLD signal increase were revealed: one located in the LFp (global maxima) and the other in the left dorso-lateral prefrontal cortex (LFdl) (Figure 2). There were also small clusters of BOLD increase in the contralateral frontal cortex and in the ipsilateral temporo-parietal cortex.

### Dynamic causal modeling analysis

Given the complexity of this case, we reviewed retrospectively the pre-surgical EEG-fMRI analysis and we decided to apply DCM to assess the effective connectivity between the two frontal clusters revealed by the pre-surgical fMRI study. Particularly we aimed to assess the causal relationship between the two clusters in relation to IED, i.e., which region drives which. We focused on the investigation of the epileptic focus [i.e., the Irritative Zone (IZ)] instead of the propagation pathways. The definition of model space was based on this primary question and on the information we already knew about the patient’s clinical history. Accordingly, two regions of interest (ROIs) were selected: the LFdl and LFp. For each ROI we computed the first principal eigenvariate of the voxel time series. The regional responses whitened and the nuisance effects were removed to obtain the corrected time courses for each region. DCM was performed using the DCM10 module as implemented in SPM8.

**Figure 3:**
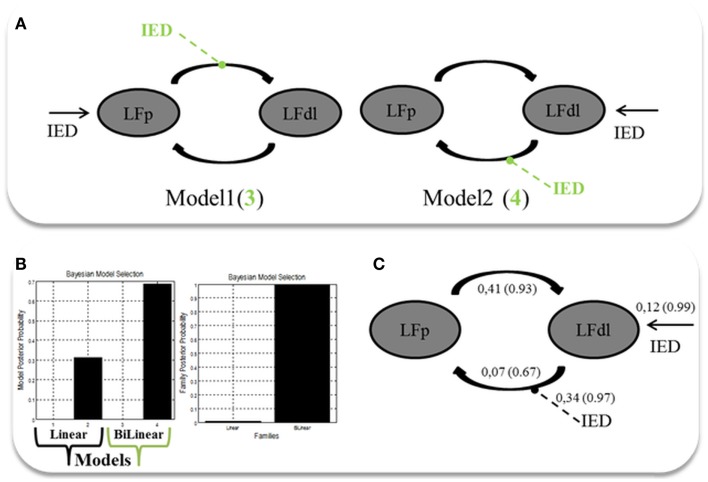
**Two ROIs effective connectivity (DCM) models**. **(A)** Two ROIs (5 mm radius) are structurally (forward and backward) connected: the left dorso-lateral prefrontal cortex (LFdl) (MNI coordinates: −48, +48, −6) and the left fronto-polar cortex (LFp) (MNI coordinates: −5, +70, +2). IED were considered as autonomous input to each of the two regions, one at a time. Two alternative competing hypotheses were then tested: (1) LFp neuronal activity drives the changes in the LFdl; (2) LFdl neuronal activity drives the changes in the LFp. For each of these connectivity structures, two types of connectivity models were then considered: the linear models (presented in black); and the bilinear models, which had linear and bilinear terms (the intrinsic connections are shown in black, the connections’ modulation in dashed green arrows). **(B)** DCM Bayesian model selection results. Left panel: relative Log-evidence for the four models compared using FFX BMS. Models are separated into Linear (Black Arrow) and Bilinear (Green Arrow). Right panel: DCM FFX family inference results according to model linearity. **(C)** Parameter averaging results. Averaged parameters obtained by FFX BPA (Bayesian Parameters Averaging) for Model 4, the best model according to BMS. Averaged modulation parameters are indicated with dashed arrow and both intrinsic connectivity and the averaged direct input parameters are indicated with solid arrows. The coupling strength of each connection is expressed in terms of Hertz (i.e., the change in neuronal activity per second as a function of inputs from other regions) with related probabilities in brackets.

Two alternative competing hypotheses were then tested: (1) LFp neuronal activity drives the changes in the LFdl; (2) LFdl neuronal activity drives the changes in the LFp. For each of these connectivity structures, two types of connectivity models were then considered: the linear models, which had only linear terms (*A* Parameters); and the bilinear models, which had linear and bilinear terms (*A* and *B* parameters). A total of four models were then compared. Each model was constituted by the two ROIs fully intrinsic connected (backward and forward): LFp neuronal activity drives the changes in the LFdl (Model 1, linear); LFdl neuronal activity drives the changes in the LFp (Model 2, linear); LFp neuronal activity drives the changes in the LFdl and IED modulates the connection from LFp to LFdl; (Model 3, bilinear); LFdl neuronal activity drives the changes in the LFp and IED modulates the connection from LFdl to LFp (Model 4, bilinear). See Figure 3A for graphical representation of the models. Fixed Effect (FFX) Bayesian Model Selection (BMS) was used to compare the individual model over the three BOLD sequences of interest. Secondly, a FFX family inference was performed by grouping the models according to model’s linearity (linear versus bilinear).

The FFX BMS results are presented in Figure 3B. The winning model was Model 4 (*p* = 0.70) following by Model 2 (*p* = 0.31). The log-evidence difference between these two models was <3 (hence not significant), while both of them were strongly more likely than Model 1 and Model 3. Both Model 2 and Model 4 are consistent with the hypothesis that the trigger of the pathological activity (IED) was the Left Fdl cortex. FFX family inference results are presented in Figure 3B: in terms of model linearity the results provided “strong” evidence in favor of the family with bilinear models (*p* = 0.99) relative to its linear counterpart, suggesting that IED modulates the strength of connections between nodes. Regarding the inferences on model parameters, the winning model FFX BPA are shown in Figure 3C.

**Figure 4:**
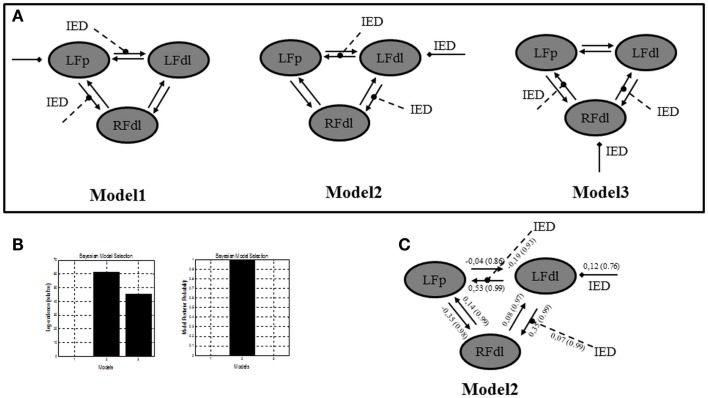
**Three ROIs effective connectivity (DCM) models**. **(A)** Effective connectivity (DCM) models. Three ROIs (5 mm radius) are structurally (forward and backward) connected: the left dorso-lateral prefrontal cortex (LFdl) (MNI coordinates: −48, +48, −6), the left fronto-polar cortex (LFp) (MNI coordinates: −5, +70, +2), the right dorso-lateral prefrontal cortex (RFdl) (MNI coordinates: +48, +48 −6). IED were considered as autonomous input to each of the three regions, one at a time. Three alternative competing hypotheses were then tested: Model 1. LFp neuronal activity drives the changes in the LFdl and RFdl and IED modulates the connection strength from LFp to LFdl and from LFp to RFdl; Model 2. LFdl neuronal activity drives the changes in the LFp and RFdl and IED modulates the connection strength from LFdl to LFp and from LFdl to RFdl; Model 3. RFdl neuronal activity drives the changes in the LFdl and LFp and IED modulates the connection strength from RFdl to LFdl and from RFdl to LFp. **(B)** DCM Bayesian model selection results. Left panel: relative Log-evidence for the three models compared using FFX BMS. The winning model was Model 2 with a log-evidence difference >3 respect with the other two models. Right panel: models Posterior Probability. Model 2 is strongly more likely (*p* = 1.00) than Model 1 and Model 3. **(C)** Parameter averaging results. Averaged parameters obtained by FFX BPA (Bayesian Parameters Averaging) for Model 2, the best model according to BMS. Averaged modulation parameters are indicated with dashed arrow and both intrinsic connectivity and the averaged direct input parameters are indicated with solid arrows. The coupling strength of each connection is expressed in terms of Hertz (i.e., the change in neuronal activity per second as a function of inputs from other regions) with related probabilities in brackets. With respect to the intrinsic connections, characterized by the linear *A* parameters, the values of the average parameters suggested that the strength of connections is enhanced in the directions LFdl to LFp and RFdl to LFp and it is diminished in the opposite directions LFp to LFdl and LFp to RFdl.

In a further analysis, we used DCM in order to test the location of the epileptic focus within different models, which included, behind the two ROIs already selected, a third region, the right dorso-lateral prefrontal cortex (RFdl). Such region is part of the epileptic network as revealed by the GLM, but from a clinical prospective (i.e., patient’s electro-clinical and neuroimaging features) it should represent an area of epileptic activity propagation instead of the epileptic focus, although this hypothesis could not be completely excluded (the presence of bilateral frontal IED on scalp EEG). By including this area in the effective connectivity analysis, we wanted to confirm, using more complex models’ architectures, the findings revealed by the previous two ROIs DCM analysis. A FFX BMS was used to compare the individual model over the three BOLD sequences of interest. A graphical description of these models can be found in Figure 4A. The FFX BMS and BPA results are presented in the Figures 4B,C respectively. The results replicated the one obtained with only two ROIs: Model 2 (LFdl neuronal activity drives the changes in the LFp and RFdl and IED modulates the connectivity strength between LFdl to LFp and LFdl to RFdl) is more likely than the other models (the log-evidence difference was >3). This indicates that Model 2 is, with “strong evidence” (*p* = 1.00), the best model explaining the data.

### Psycho-physiological interaction analysis

**Figure 5:**
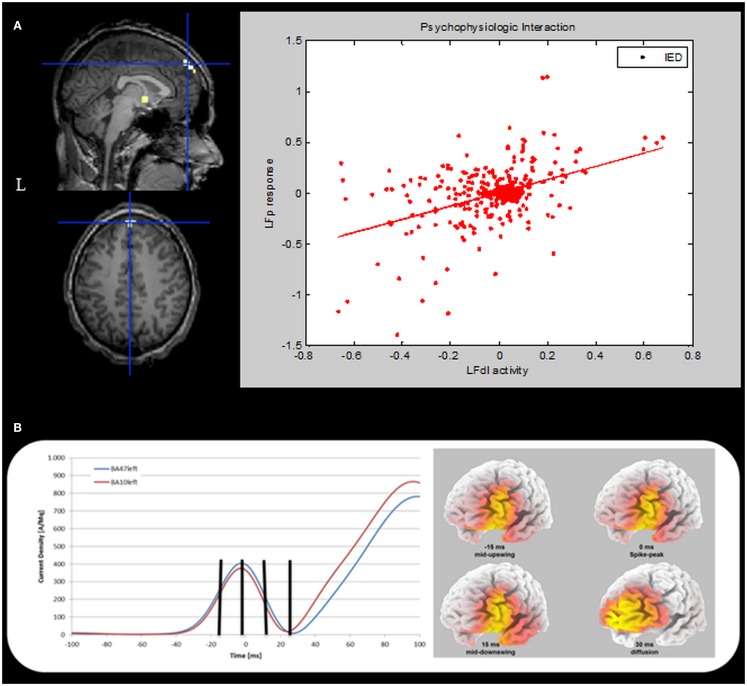
**Psycho-physiological interaction and ESI results**. **(A)** PPI results. Left panel. SPM{T} (*p* < 0.05 corrected for multiple comparison) showing isolated BOLD signal increases in the left medial frontal gyrus (global maxima) (BA10, MNI coordinates: −1, +66, +26). This cluster shows a positive correlation with the LFdl during IED. Results were overlaid on the pre-surgery patient’s T1 scan. L, Left; BA, Brodmann area. Right panel: scatterplots and regression line of the LFp-LFdl correlation in relation to IED. The line corresponds to the regression. IED can be seen to augment the contribution of LFdl to LFp activity. This regression demonstrates the contribution of the interaction effect to LFp response and can be interpreted as evidence for a positive modulation of LFdl to LFp by IED. **(B)** ESI results. Left panel: the mean current density was computed for all voxels belonging to left BA10 (red line) and left BA47 (blue line). The vertical black marks indicate the same time points reported in the right panel. Right panel: current density distribution evaluated at four different timings: 15 ms before spike peak (mid-upswing), spike peak itself, 15 and 30 ms after the spike peak. BA, Brodmann area.

To further validate our findings, a confirmatory Psycho-Physiological interaction (PPI) analysis was performed. PPI provides information about the way in which activity in one brain region modulates activity in another brain region specifically in response to the active task relative to the baseline or another task (4). In the case of the current study, PPI can be used to test if activity in the LFp cortex is predicted on the basis of activity in LFdl in relation to IED. To perform PPI analyses the individual first eigenvariate time series from a sphere of 5-mm radius (physiological variable), centered on the left dorso-lateral prefrontal cortex was extracted. A second regressor representing the experimental condition (in our case the IED) was entered in the analysis as the psycho-physiological variable. The interaction between the experimental condition and the seed region activation signal (the PPI) was chosen as regressor of interest for the PPI analysis. One-tailed *t*-test was applied to test for positive and negative PPI. The computed SPM{T} was thresholded at *p* < 0.05, corrected for multiple comparisons. The PPI results are shown in Figure 5A. There was only one region showing a positive correlation with LFdl cortex in relation to IED: it was the left frontal anterior cortex, particularly the global maxima (which survives at the corrected threshold) is located in the left medial frontal gyrus (Brodmann Area 10). This finding demonstrates the contribution of the interaction effect to LFp response and can be interpreted as evidence for a positive modulation of LFdl to LFp by IED. No negative correlations with LFdl were detected.

### Electric source imaging analysis

Finally, we performed an Electric Source Imaging (ESI) of the IEDs recorded during the pre-surgical EEG-fMRI study: 90 IEDs were identified and marked; segments from 100 ms before the spike peak to 100 ms after the event were aligned and averaged; the source reconstruction analysis was performed using sLORETA algorithm (5). ESI revealed a main source in the left dorso-lateral frontal cortex (left inferior frontal gyrus – BA47; best fit at MNI coordinates: −25, +35, −5) over the most of the spike time (from −28 to +27 ms with respect to the peak of the averaged IED), including the mid-point of the ascending phase, which is considered to reflect the epileptic focus localization most reliably (6). Interestingly, involvement of the frontal pole (left medial frontal gyrus-BA10; best fit at MNI coordinates: −5, +45, −10) was evident around 30 ms later at the slow-wave onset (Figure 5B).

### Validation of non-invasive techniques by intracranial EEG recording

**Figure 6:**
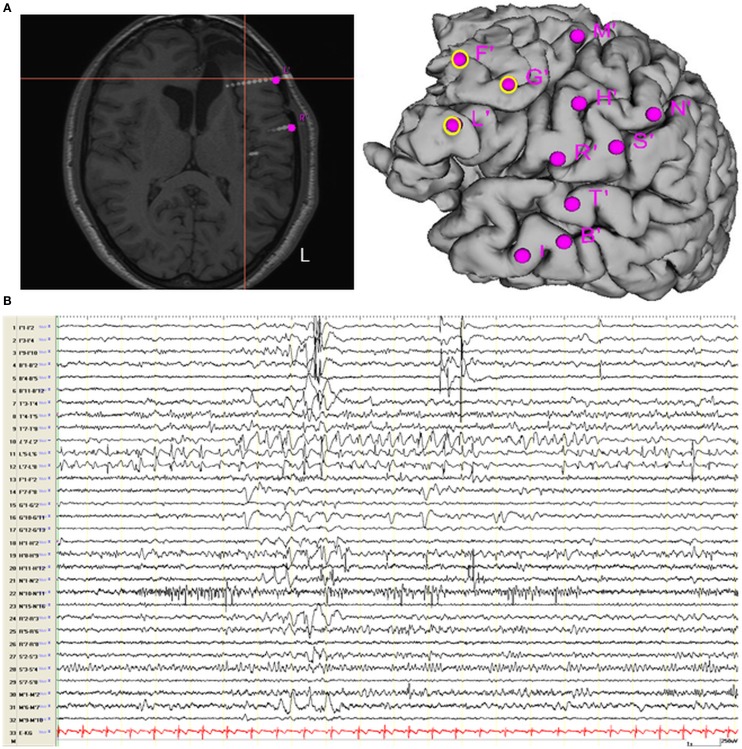
**Intracranial EEG recording recording**. **(A)** Localization of SEEG electrodes shown on patient’s T1 scan (slice and cortical rendering). The yellow ring over the cortical subject’s surface identified the seizure onset zone. Electrodes locations: L (lesion), R = inferior frontal gyrus; F, G = middle frontal gyrus; M = superior frontal gyrus; N, S = motor cortex; T = superior temporal gyrus; B, I = middle temporal gyrus and hippocampus. **(B)** Interictal Stereo-EEG (SEEG) recording shown continuous interictal epileptic paroxysms over the electrodes “L” and less pronounced on the electrodes “F” and “G.”

**Figure 7:**
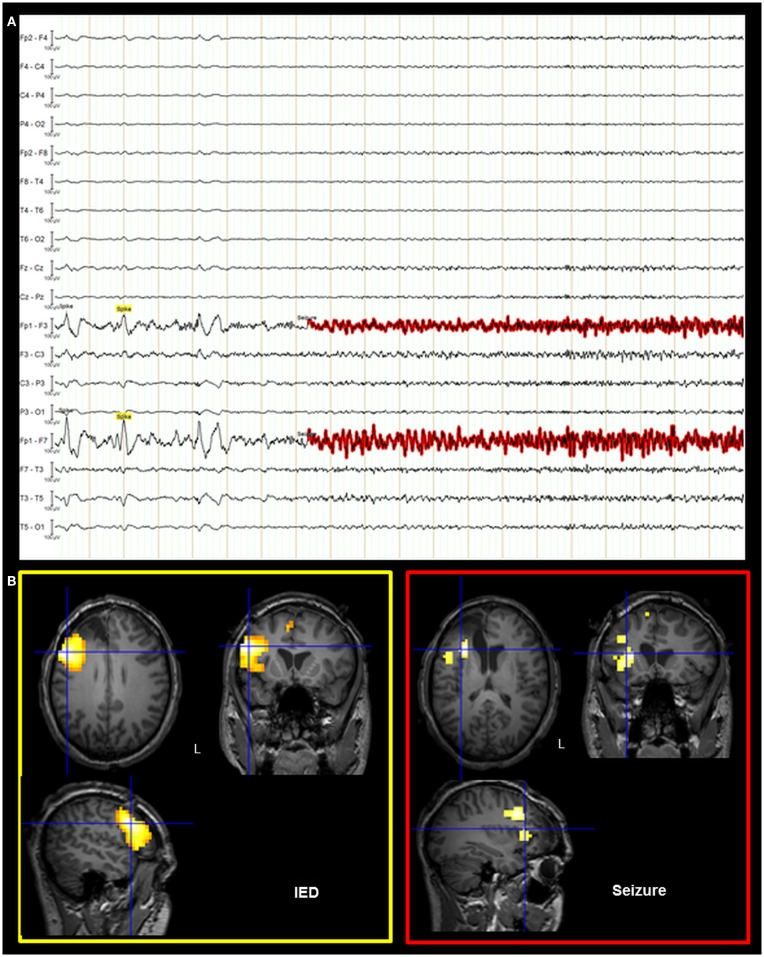
**Post-surgical EEG/fMRI results**. **(A)** Representative page of the EEG recorded during fMRI data acquisition. The EEG trace is shown in bipolar montage: 205 IED (underlined in yellow) were recorded characterized by spikes and sharp-waves located over the left frontal regions; a single sub-clinical seizure (underlined in red) was acquired, showing low-voltage fast (around 14 Hz) activity on the left anterior frontal leads (Fp1, F3, and F7 electrodes on scalp EEG). **(B)** Results of canonical GLM, SPM{T} (*p* < 0.05 corrected for multiple comparison) showing BOLD signal increases IED and seizure related. Crosshair at the global statistical maximum: an isolated cluster was observed at the left dorso-lateral frontal cortex (BA47). Results were overlaid on the post-surgery patient’s T1 scan. L, Left; BA, Brodmann area.

On the basis of the consistent fMRI maps, the DCM and ESI results we hypothesized that the ictal onset zone was located over the left dorso-lateral frontal cortex. This hypothesis was indeed finally validate by the icEEG monitoring performed by the patient after the surgery failure. Eleven electrodes were implanted (at the “C. Munari” Center for Epilepsy Surgery, Milan) according to the stereo-EEG (SEEG) methodology exploring the patient’s left frontal and temporal lobe (Figure 6A). The icEEG demonstrated a SOZ involving the electrodes located over the left pre-motor dorso-lateral frontal cortex. In particular, 40 seizures were recorded during icEEG monitoring (Figure 6B), all characterized by an ictal discharge over L’, F’, G’ contacts (yellow circles in Figure 6A). The same contacts disclosed a sub-continuous spike activity typical of FCD (7). Moreover, a second EEG/fMRI study was performed at this time using the same scanner and procedure, showing frequent left frontal IEDs and a 30-s sub-clinical seizure, characterized by low-voltage fast activity (14 Hz) over the left frontal area (electrodes Fp1-F3-F7) (Figure 7). fMRI data analysis demonstrated a single region of BOLD signal increase in the LFdl cortex related to the IEDs and ictal discharge, respectively.

The SOZ delineated by icEEG totally overlapped with the BOLD signal changes revealed by the two EEG-fMRI studies. A second cortectomy including the left dorso-lateral frontal cortex was then proposed to the patient after performing a language-fMRI study that confirmed a right-hemisphere dominance (data not shown). Unfortunately, the intervention was precluded by an anaphylactic reaction to anesthetic drug.

## Background

Simultaneous recording of EEG and functional MRI (EEG-fMRI) is a technique capable of revealing the brain regions involved by the epileptic discharge based on local BOLD signal variations. In patients with focal epilepsy the significant clinical question is how the EEG-fMRI results can contribute to localize the SOZ, the brain region that is thought to be responsible for generating seizures. icEEGs are considered the gold standard for the validation of the EEG-fMRI studies (8).

Recent work has demonstrated a significant contribution of the interictal epileptic discharges (IED)-related BOLD changes in localizing the brain regions that give raise to IEDs (9), and in post-surgical populations it was shown that when the resection included the IED-related BOLD clusters, patients showed good outcome (10). This evidence further supports the importance of a correct definition of IED generators in order to improve surgery outcome.

The concept of epileptogenic networks, in contrast to a single region giving rise to seizures, has been proposed (11). Diagnostic methods based on linear and non-linear regression analysis of icEEG signals have been employed to characterize the functional connectivity within the epileptic network and identify the drivers of the pathological activity (12, 13). Such methods suggested that the epileptogenic zone is organized as a network of distinct- and possibly distant-neuronal networks with altered excitability properties and abnormal facilitated connections (14). There is therefore great interest in better identifying the nodes of such networks and the inter-relationships between nodal activities.

In the recent years, new techniques have been developed to address the connectivity in epilepsy based on the application of MRI. There are two state-of-the-art approaches for understanding the communication among distributed brain system using fMRI: functional and effective connectivity analysis. Both of them are aimed at identifying the presence and the strength of connections between network nodes and when possible, their directionality (15). However, compared to functional connectivity approaches, a further ambition of effective connectivity is to allow the inference of (biophysical) mechanisms by which causal links are expressed in measured neuroimaging signals (16). It means that the study of effective connectivity is usually more model-based (or hypothesis driven) than that of functional connectivity (17). Within effective connectivity methods, DCM on fMRI data is an innovative approach, which could provide information about the causal interactions among neuronal states (18) and hence potentially might identify the neuronal drivers of pathological activity. This implicates that valid inference can be made about, for example, which brain region drive which, despite the limitation of temporal resolution inherent to fMRI. In epilepsy field, in which the identification of the neuronal drivers of pathological activity is crucial for patient management, DCM represents an innovative and potentially revolutionary approach of neuroimaging data analysis. In brief, DCM for fMRI data combines a model of neural dynamics within experimentally validated hemodynamic model that describes the transformation of neuronal activity into a BOLD response (18–20). Both sets of parameters describing the neuronal state and those determining the forward model of BOLD signal generation are estimated from the data within a Bayesian framework for each brain area included in the model (21). Hence, crucially, the possibility for differing hemodynamic responses (e.g., latency between regions) is included within the DCM. The Bayesian framework allows an inference to be made as to whether the data is best explained by variations in the hemodynamic response or instead by changes in the underlying neural system.

To date, only a few studies applying DCM on fMRI data in epilepsy have been published (22–26). Among these, the most far-reaching experimental assessment of the validity of DCM analysis was done by David et al. (22), who performed concurrent fMRI and icEEGs to measure the spread of excitation in a genetically rat model of absence seizures. This allowed them to infer the connectivity using just the fMRI data (with DCM) and compare the estimates to the true connectivity based on electrophysiology using intracranial recordings. In human epilepsy, Hamandi and colleagues published the first study that applied DCM on fMRI data in 2008 (23). The authors wanted to assess the effective connectivity between brain regions, namely parahippocampal gyrus and lingual gyrus, activated during interictal spikes in a patient affected by temporal lobe epilepsy (TLE). DCM analysis revealed a propagation of neural activity from the temporal focus (the IZ) to the area of occipital activation (lingual gyrus). More recently, the same approach was used to study the propagation pathways of the seizure activity recorded in a patient with hypothalamic hamartoma (26). Both these studies were focalized on the investigation of epileptic activity propagation from a known focus toward an extended brain network. This information might significantly contribute in the decision of the surgical approach for epilepsy treatment. Of similar importance and even more, is the recognition of the brain focus generating the ictal and interictal activity (i.e., the SOZ and IZ). Attempts to reach this objective have been recently published by using DCM applied on fMRI recorded in patients with generalized and partial epilepsy (24, 25). All the described works did not provided a validation of the DCM results by means of the icEEG recordings in term of the epileptic trigger and propagation pathways as instead performed in animals (22). Therefore, such validation study in humans has not been reported to date.

## Discussion

We studied a case of sub-optimal post-surgical outcome to evaluate the potential clinical role of advanced analysis of non-invasive procedures by re-analyzing pre-surgical EEG and fMRI data. Our findings underline the importance of a careful interpretation of all pre-surgical imaging and electrophysiological data using the most advanced analysis approaches in order to obtain a better patient outcome.

Particularly, this work represents the first attempt to validate DCM results on the effective connectivity of networks involved during IED in a patient affected by focal refractory epilepsy. Two competing hypotheses on the causal network involved during IED were tested based on the clinically plausible scenario and the GLM analysis of pre-surgical fMRI data.

The main result of this study is that the effective connectivity analysis performed on the pre-surgical fMRI data was able to identify a causal link from the dorso-lateral cluster to the fronto-polar one, suggesting that the latter represents an area of IED propagation. This finding is supported by PPI results, which added evidence for a positive modulation of LFdl to LFp by IED. Although both analyses have given concordant findings, it is remarkable that, for the intrinsic differences between the two methods, DCM provides more robust statements about effective connectivity and causality (27). Similarly to the effective connectivity analyses, ESI revealed a pattern of spike propagation from the dorso-lateral frontal cortex to the fronto-polar region. Our results corroborated the use of a multi-modal approach to investigate the epileptic networks (28).

To our knowledge only another two reports have evaluated the usefulness of DCM in the context of symptomatic focal epilepsies (23, 26). Our work is the first that has evaluated the DCM results in relation to the surgical outcome and the icEEG findings, both supporting the validity of the connectivity analysis.

EEG-fMRI is increasingly being used in the epilepsy centers to help localize epileptic activity (29). An important clinical limitation of EEG-fMRI resides in the interpretation of multiple clusters of BOLD signal changes: which one(s) represent(s) the site of IED/seizure origin and which are involved due to propagation? In our case, indeed, the conventional fMRI analysis was unable to identify which cluster (or both) must be removed to obtained the seizure freedom. Our findings suggest that DCM of fMRI may be a useful tool to assess the causal hierarchy within epileptogenic networks. The characterization of the epileptic network and especially the ability to identify the driver of the pathological activity would improve the patient’ assessment by assisting the surgeon in achieving “optimal” delineation of the volume of tissue to be excised. Necessarily, these results should be interpreted with caution. Firstly they refer to a single case and the validation of the DCM analysis was performed after surgery failure. Secondly, our conclusions are valid solely with respect to the family of tested models. Theoretically, there may be brain areas, which are involved in the IED generation processes that were overlooked because of their apparent lack of hemodynamic involvement.

We have assessed the causal hierarchy within a simple connection models involving only two brain regions. The interactions, occurring during partial seizures and interictal activity generation, are usually more complex and might involve areas distant respect with the presumed SOZ (9, 12). However, in our case, the two competing hypothesis on IED generation were both strongly physiological plausible and the specified two nodes allowed us to test them. Our approach is in line with the premise that DCM should be used to test specific hypothesis rather than an exploratory one (18).

An interesting aspect of this work is that DCM analysis on fMRI, PPI analysis, and ESI on EEG data showed similar results with respect to the putative SOZ. Clinically, this finding is relevant because all these approaches if performed before surgery could have driven an icEEG recording allowing a wider frontal resection, including the dorso-lateral cluster, and hence a potential better patient outcome. Of course, we could not assess, with certainty, if the resection of the dorso-lateral frontal cortex alone would have been sufficient to obtain seizure freedom, since the presence of a FCD over the fronto-polar area suggests a possible intrinsic epileptogenicity also of this region.

In the end, the inability to perform a second operation for removal of the dorso-lateral frontal cortex prevents a definitive proof that the SOZ is actually within this region. However, we believe that the concordance of different non-invasive techniques with each other and in comparison with the results of icEEG recordings supports, clearly, the hypothesis of the dorso-lateral frontal cortex as the SOZ. Furthermore, clinically speaking, the persistence of seizures with an identical semiology after the first operation is a proof that the dorso-lateral cortex did not represent merely a region of propagation of the discharge, but at least the region co-participant in the genesis of the seizures.

From a methodological point of view, the observation that ESI replicated DCM findings is intriguing and further validate the effective connectivity analysis based on fMRI data, despite the much slower temporal resolution (seconds) of BOLD signal compared to electrophysiological measurements (milliseconds). Simultaneous ESI with fMRI suggests that EEG-derived BOLD maps represent epileptic network activity reflected in the EEG (30) and the combination of the two techniques allows a better identification of areas of IED initiation from regions of propagation (31).

### Methodological considerations

One limitation of our study is that the implementation of DCM used presumes the interictal activity as an extrinsic input, which is obviously might be argued for such an endogenous type of activity. The knowledge of an input which enters and perturbs the system is required as DCM was conceived based on extrinsic inputs under experimental control. In the approach used here, the IED were conceived as a time marker of an event taking place within the epileptic focus and which perturbs the postulated network. The time of IED onset is hence assumed to be the initial cause of the modeled effects as it can influence directly the neuronal states of the specified anatomical nodes. Similar to previous studies (22–24, 26), we have considered the system’s input as a block or a stick function corresponding to the periods of interictal activity identified on the EEG. This approach has its limitation as the time of IED was derived by simultaneous scalp EEG which might be delayed respect with the real interictal activity onset (32). Additionally, a stick function or a single-block almost certainly do not represent dynamic processes such as the epileptic activity are (33). The recent developments of stochastic DCM (sDCM) (34, 35) may provide more suitable approaches for modeling spontaneous epileptic activity. A pioneer study in this contest has been recently published which represented a validation of sDCM for fMRI data in relation to electrophysiological responses (36).

## Concluding Remarks

This paradigmatic case shows how EEG-fMRI combined with multi-modal approaches of fMRI data analysis may give useful information to identify the SOZ and propagation patterns of epileptic activity. Of course, further prospective studies are required to assess the role of this non-invasive tool in the diagnostic work-up of patients with surgically remediable epilepsies. In particular, this case-study underscores the importance of a multi-modal approach to the analysis of EEG and fMRI signals to better characterize the epileptic network and its intrinsic connectivity.

## Author Contributions

Anna Elisabetta Vaudano, Pietro Avanzini, Laura Tassi, Andrea Ruggieri, Francesca Benuzzi, Gaetano Cantalupo contributed to data acquisition and data analysis. Anna Elisabetta Vaudano, Pietro Avanzini, Paolo Nichelli, and Stefano Meletti wrote the article. Anna Elisabetta Vaudano and Stefano Meletti, were involved in conception, and interpretation of the data presented.

## Conflict of Interest Statement

None of the authors has any conflict of interest to disclose. A Ph.D. bursary from the University of Modena and Reggio Emilia supported Andrea Ruggieri. Anna Elisabetta Vaudano and Pietro Avanzini are supported by a post-doc grant from the “Fondazione Cassa di Risparmio di Modena.” We confirm that we have read the Journal’s position on issues involved in ethical publication and affirm that this report is consistent with those guidelines.
